# Equity of the premium of the Ghanaian national health insurance scheme and the implications for achieving universal coverage

**DOI:** 10.1186/1475-9276-12-4

**Published:** 2013-01-07

**Authors:** Eugenia Amporfu

**Affiliations:** 1 Kwame Nkrumah University of Science and Technology, Kumasi, Ghana

## Abstract

The Ghanaian National Health Insurance Scheme (NHIS) was introduced to provide access to adequate health care regardless of ability to pay. By law the NHIS is mandatory but because the informal sector has to make premium payment before they are enrolled, the authorities are unable to enforce mandatory nature of the scheme. The ultimate goal of the Scheme then is to provide all residents with access to adequate health care at affordable cost. In other words, the Scheme intends to achieve universal coverage. An important factor for the achievement of universal coverage is that revenue collection be equitable. The purpose of this study is to examine the vertical and horizontal equity of the premium collection of the Scheme. The Kakwani index method as well as graphical analysis was used to study the vertical equity. Horizontal inequity was measured through the effect of the premium on redistribution of ability to pay of members. The extent to which the premium could cause catastrophic expenditure was also examined. The results showed that revenue collection was both vertically and horizontally inequitable. The horizontal inequity had a greater effect on redistribution of ability to pay than vertical inequity. The computation of catastrophic expenditure showed that a small minority of the poor were likely to incur catastrophic expenditure from paying the premium a situation that could impede the achievement of universal coverage. The study provides recommendations to improve the inequitable system of premium payment to help achieve universal coverage.

## Introduction

Universal coverage is achieved in a health system when all residents of a country are able to have access to adequate healthcare at affordable prices [1]. The achievement of such a goal requires the provision of adequate healthcare as well as the availability of a healthcare financing system that ensures access to adequate care regardless of ability to pay. In other words, the healthcare financing system required for a universal coverage has to be equitable. A healthcare financing system is equitable if household payment for healthcare is in accordance with ability to pay and utilization is in accordance with need [2]. Equity then refers to the ability of the health system to provide financial protection to all (potential) users of healthcare.

The ethical justification for healthcare equity comes from the strong link between healthcare and health. People do not desire healthcare but health. However health is not sold in the market so they need to purchase healthcare in order to produce health. Good health is needed for people to thrive as human beings [3]. Thus what drives policy makers to find it fair to link payment of healthcare with its utilization or ability to pay is the fact that healthcare payments are not voluntary item of expenditure but it is caused by unwanted health shock and that society as a whole is willing to share in absorbing the burden [4]. Alternatively, others argue that the need for equity is driven by the belief that healthcare is a right and so it is the obligation of society to ensure that everyone has access to the good regardless of ability to pay [5].

Equity in healthcare financing can be vertical (variation in financial contribution is proportional to ability to pay) or horizontal (people of the same ability to pay making the same contribution). Studies on vertical equity have focused on the progressivity of healthcare financing which refers to the tendency of healthcare payment, as a proportion of income, to rise as income rises [3]. This implies that in a progressive healthcare financing system the poor spend a lower proportion of their income on healthcare than the rich. A progressive health financing reduces financial burden on the poor in general and hence is likely to provide financial protection and access to healthcare. Horizontal equity has received less attention in the literature but is important in ensuring that financial protection is not provided only to some poor residents while other poor residents are left without protection. The reason is that horizontal inequity could imply that some poor people make higher healthcare payment than other poor people.

Inequity in healthcare financing can cause financial burden through the effect on income redistribution. Income redistribution that results from healthcare financing can be due to vertical or horizontal equity/inequity, and/or income re-ranking. Income re-ranking occurs when income after healthcare payment moves the payer into a different income group. Progressive and horizontally equitable healthcare payment can have a positive effect on income distribution by reducing any preexisting inequity in income distribution. Thus both types of equity are important for universal health coverage. The purpose of this paper is to use new survey data to measure the vertical and horizontal equity of the premium contribution of the Ghanaian National Health Insurance Scheme (NHIS) and the implication for universal coverage. Even though the interest of this study is in the two types of equity, income re-ranking is included in the analysis because the model used for the analysis is unable to separate horizontal equity from income re-ranking.

### The Ghanaian national health insurance (NHIS)

In order to improve equity in healthcare financing and utilization, the NHIS was introduced in 2003 to replace an existing user fee system locally known as the Cash and Carry System. Under the Cash and Carry system, patients had to pay for healthcare services and so utilization was skewed in favor of the rich. Certain exemptions measures were put in place to cushion the negative effect of user fees on the poor but these measures did not work due to poor implementation and unclear guidelines. Thus there was no financial protection available for the poor, and healthcare financing was highly inequitable. Since everybody paid the same fee for a given service, and a given fee was likely to form a greater proportion of the income for the poor than the rich, the cash and carry system was regressive. Even though the flat fee for a given service was borne by all and so one would expect that people of the same income would pay the same fee for the given service, the variation in the probability of being sick within a given income group implies that the high risk in the group were likely to spend more on healthcare than the low risk. Hence, the Cash and Carry system was both vertically and horizontally inequitable.

The NHIS is a social insurance scheme with the vision of providing financial risk protection for basic quality health care for its members. Membership to the scheme is currently voluntary and according to the NHIS report for 2009, enrolment to the scheme is 62% of the Ghanaian population. The NHIS raises revenue from six sources: the health insurance levy (67%), insurance premiums (5.0%), Social Security of National Insurance Trust (SSNIT) contributions (15.6%) investment income (17%), sector budget support (2.3%), and other sources (0.2%) [6]. Studies have examined the progressivity of the health insurance levy which is 2.5 percentage of an existing Value Added Tax and have concluded that it is progressive [7,8]. However [7], showed that while the overall financing of the NHIS was progressive, the premium revenue was regressive. In a recent study [9], found that the health insurance levy is mildly progressive but the premium revenue still regressive. Equity of the premium is important in ensuring membership because residents may have to pay premium in order to register with the NHIS. If the premium imposes financial burden on existing or potential members, membership could fall and hence impede the ability of the NHIS to achieve universal coverage.

To minimize the potential financial burden that could be imposed by the premium several categories of members are exempt from premium payment. For example, to ensure coverage for children and to reduce maternal and child morbidity and mortality, children under eighteen years of age (whose parents are registered with the NHIS) and maternal patients are exempt from paying premium. These categories of members form about 54.9% of the registered members. The maternal exemption category was made possible by a grant from the government of UK. Indigents and members above seventy years of age are also exempt from premium payment. Adults in the formal sector who make pension contributions to the Social Security and National Insurance Trust (SSNIT) and SSNIT pensioners do not pay premium. Only adults in the informal sector who form about thirty percent of the membership pay premium. Thus thirty percent of the members pay the premium which contributes only 5.0% of the total revenue. The scheme then relies on the other sources of funds for operation.

Even though the premium revenue forms only a small percentage of the total revenue that goes to the NHIS, the premium is a very important determining factor for the achievement of universal coverage. According to a recent study [10], the poor are less likely to enroll with the NHIS than the rich. This could affect the ability of the poor that are currently enrolled on the scheme to remain on it. Given that the percentage of indigents in Ghana is 18 [10], the formal sector employs about 10% of the workforce [11] (implying that 90% is employed by the informal sector), about 50.5% of the population form the workforce [12], and assuming that the indigents are not part of the workforce it follows that about 45.5 (0.9 * 0.505) percent of the population would pay premium if the whole Ghanaian population were enrolled with the scheme. Thus given the current Ghanaian population of about 24.9 million, there are about 3.6 million adults plus their children who are not enrolled in the scheme. High premium could be one of the reasons for their lack of participation in the scheme. Focusing mainly on the revenue contribution of the premium could make one miss the important role played by the premium in determining membership and hence the sustainability of the scheme. The importance of equity of the premium then cannot be overemphasized.

The NHIS is made up of District Mutual Health Schemes (DMHS) regulated by the National Health Insurance Authority. Registered patients pay no fee at the point of purchase of healthcare. Members register/renew their membership and pay their premium at the DMHS. Premiums range between GHc 7.2 and GHc 48 (US$4.8 and US$32) according to ability to pay. There is no clear guideline regarding how much is to be paid according to a given level of income. In addition, the large informal sector makes it even difficult for the DMHS to have access to information on the incomes of registered members. Often, a flat premium is charged to all members of a given district mutual health scheme but adjustments could be made when information on area of residence, household size, etc. are revealed.

With a gini index, of 0.40 [13], income distribution in Ghana is highly inequitable. Even though the extent of the inequality is less than that in South Africa, it is worse than that of Tanzania [14]. The significant disparity of income implies that premium payment should also vary significantly to ensure equity in premium contribution. Since the DMHS that determine the premium contributions of members do not have information on members’ income, it is possible for members of equal income and household size to pay unequal premiums. With high asymmetric information on income in the informal sector where the poor are likely to be found, it is possible for the premium contribution of the NHIS to be vertically and/or horizontally inequitable. The question is: to what extent does any existing inequity impose financial burden on the poor and hence impede the achievement of universal coverage.

### Universal health coverage and equity

As indicated in the definition, universal coverage is achieved when all residents have financial protection from health expenditure and have access to adequate healthcare. This implies that universal coverage leads to equity. Universal coverage has thus been advocated as a means of achieving health equity. Existing literature have thus shown how the achievement of universal coverage has led to equity in the quality of care. For example [15], showed that after the introduction of the universal coverage program, healthcare financing inequity in Thailand reduced as a result of a continual reduction in catastrophic expenditure. Others have argued that if the approach of universal coverage does not start with coverage for the poor, but focuses on those that are easiest to reach, there is the possibility of initial inequity as the rich get better access to healthcare while the poor wait to get the trickling down benefits [16]. Countries like Brazil and Mexico that targeted the poor to ensure that the poor receive at least the same benefit as the rich, i.e., progressive universalism, are likely to avoid the initial inequity between the rich and poor [16]. The Ghanaian NHIS is accessible to the poor as long as the premium contribution does not impose financial burden on the poor.

For universal coverage to be achieved through the NHIS all Ghanaian residents, regardless of economic status, are to register with the scheme. This can happen if new members join and/or the existing members do not leave. When the revenue collection is vertically inequitable it might impose financial burden on the poor in general and discourage them from remaining with the scheme. Hence, vertical equity is important for universal coverage. Even though horizontal equity has not received any attention in the literature regarding the equity of the NHIS, horizontal equity must also be important in ensuring universal coverage. The reason is that even if the payment is progressive but horizontally inequitable, it is possible for the payment to impose financial burden on some of the poor and hence discourage them from remaining or joining the scheme.

Horizontal inequity can also taint the public image of the NHIS and reduce membership and hence prevent or slow down the achievement of universal coverage. This is because while it is difficult for a low income earner to determine that higher income earners are paying disproportionately smaller percentage of their income (i.e., contributions are regressive), it is much easier to observe inequity in the premium payment among people of the same income group. Thus inequity in premium contributions by people in the same income group could displease members and hence reduce membership unless the criterion for the variation is socially acceptable.

Universal coverage can still be achieved even in the presence of vertical and horizontal inequity, if the premium payment does not lead to catastrophic expenditure. If the premium is very small relative to peoples’ ability to pay then it will not cause financial burden even on the poor and all can receive financial protection. Thus in addition to finding the equity of the premium, the study also examined the tendency of the premium to cause catastrophic expenditure. To the author’s knowledge no such comprehensive study has been done on the premium of the NHIS.

### Ability to pay variable

In the formal sector, income level is used as indicator of ability to pay or welfare. Income is regularized and verifiable in the formal sector and so it is a good indicator of welfare. In the informal sector however income is irregular and so studies often use household consumption expenditure as a measure of ability to pay or welfare. This is in accordance with the permanent income hypothesis that states that people smooth their consumption overtime even if income is not regular.

Consumption expenditure as a measure of ability to pay however is not without problem. The main weakness of the measure is the implied assumption that healthcare expenditure does not affect saving decision. The use of consumption expenditure as a measure of welfare or ability to pay assumes that households with higher ability to pay have higher consumption expenditure than those with lower ability to pay. The measure then does not take into account the fact that households may have to borrow in order to increase consumption expenditure [17]. Besides the ability to pay of households that are able to produce their own foodstuff would be underestimated. An increase in consumption expenditure for a household could also be due to debt repayment and hence may not imply an increase in goods and services consumed but a fall or no change in welfare. To minimize the negative effects consumption expenditure as a measure of welfare is computed as gross household expenditures on food and non-food items including taxes, social security, as well as all out of pocket expenditures on healthcare [17]. Since the interest in the current study is in the distributional impact of the premium, the gross expenditure of households on food and all other household needs including health care expenditure was used as an indicator of ability to pay.

## Methodology

The equity analysis was threefold. The study first assessed the progressivity of the NHIS premium and measured the degree of progressivity. Second, the study computed the redistributive effect of the premium, a measure that involves the computation of the horizontal inequity. Lastly the study computed the incidence and intensity of catastrophic expenditure of the premium.

To assess the progressivity of the premium, households in the data were categorized into ability to pay quintiles. Concentration curve and Lorenz curves were drawn on the same graph for the ability to pay quintiles and compared. The concentration curve plotted the cumulative percentage of the premium contribution against the cumulative percentage of the sample according to ability to pay in increasing order. If the premium contribution was the same regardless of ability to pay the concentration curve would be equal to the 45° line. If the premium contributions of the poor exceeded that of the rich then the curve would lie above the 45 degree line, otherwise it would lie below the line. The Lorenz curve is the representation of the cumulative distribution of ability to pay among the group. If ability to pay was evenly distributed the Lorenz curve would be equal to the 45 degree line otherwise it would be convexly sloped under the 45 degree line. If the concentration curve was everywhere below the Lorenz curve then the premium collection would be progressive. The premium collection would be regressive if the concentration curve was everywhere above the Lorenz curve [17].

The method introduced by [18] was used to measure the ability to pay redistributive effect of the premium. The redistribution is measured as:

$$\begin{array}{l} \mathrm{RE}=V+H+R\\ \;=\left(\frac{g}{1-g}\right)K_{p}-\sum \alpha_{x}G_{x-p}-\left[G_{x-p}-C_{x-p}\right] \end{array}$$

where *g* is the average share of ability to pay taken up by the premium, *K*_*p*_ is the Kakwani index of premium progressivity, *α*_*x*_, weight, is equal to the product of the square of population share of those with pre-premium ability to pay *x* and post premium ability to pay share of the pre-premium ability to pay of the group, *G*_*x-p*_ is the post premium Gini coefficients of those with prepayment ability to pay *x*, *C*_*x-p*_ is the post premium concentration index which is obtained by first ranking the households by their pre-premium ability to pay and then within each equal ability to pay group rank them by their post premium ability to pay. The first term on the right hand side (*V*) measures the vertical redistribution or the inequality reduction that would occur in the absence of horizontal inequity. The second term (*H*) measures horizontal inequity and is equal to the weighted sum of the post-premium ability to pay Gini coefficients of the ability to pay groups. The third term, (*R*) measures re-ranking that occurs from the move from an ability to pay group to another as a result of the premium contribution. If *R* is zero there is no re-ranking. According to Aronson et al., *H* increases while *R* falls as the range of income used to define ‘equals’ widens. Thus a distinction between *H* and *R* is not interesting. The focus of the analysis was on the component of *V* versus *H* + *R* in income redistribution. However reranking is typically caused by horizontal inequity and so *H + R* was considered as capturing horizontal inequity [4].

In general Kakwani index equals twice the area between the premium concentration curve and the Lorenz curve and is computed as *K*_*p*_ = *C - G* where *C* is the pre-premium concentration index and *G* is the pre-premium Gini coefficient. The -2 <*K*_*p*_ < 1. If *K*_*p*_ < 0, then the premium is regressive. The study applied a convenient regression below found in [17] for the estimation of the Kakwani index:

$$2\sigma_{R}^{2}\left[\frac{h_{i}}{\eta }-\frac{y_{i}}{\mu }\right]=\alpha +\beta R_{i}+e_{i}$$

where *R* is the fractional rank of the ability to pay variable, *σ*_*R*_^2^ is the variance of *R*_*i*_, *h*_*i*_ is the premium paid by household, *η* is the mean of premiums paid, *y*_*i*_ is the ability to pay variable and *μ* is the mean of ability to pay. Kakwani index is the coefficient of *R*. The formula for Gini coefficient is: $G=1-\frac{\frac{2}{T}\left(\sum_{i=1}^{n-1}x_{i}\right)+1}{n}$ where *x*_*i*_ is the cummulated values of the premiums for individuals, *T* is the last value of the cummulative column, and *n* is the sample size. Gini coefficient is between zero and one with zero meaning perfect equity and one implying all income is owned by one person and nothing for others (perfect inequity). A convenient regression below, also from [17] was used to estimate the Concentration Index:

$$2\sigma_{R}^{2}\left[\frac{h_{i}}{\eta }\right]=\vartheta +\gamma R_{i}+\varepsilon_{i}$$

where the *γ* is the concentration index and all other variables are as defined above.

Following [17], catastrophic expenditure was computed as the ratio of the premium and the ability to pay variable: *P*_*i*_*/y*_*i*_ where *P*_*i*_ is the premium for household i and *y*_*i*_ is the household’s ability to pay variable. A household is said to incur catastrophic expenditure if the ratio exceeds a threshold, *z*. Following the literature, a range of values were used as threshold: *5, 10, 15*, and *20*%. For example, a threshold of 10% means that if the premium payment forms more than 10% of the household expenditure on food and others the premium payment causes household to sacrifice essential needs, sell assets, or become impoverished. Where there is catastrophic expenditure, information on incidence and the intensity of the catastrophic expenditure would be important. The incidence simply means the number of households who incurred catastrophic expenditure for a given threshold. It is computed as the fraction of the households who incurred catastrophic expenditure: $H=\frac{\sum_{i=1}^{N}E_{i}}{N}$*N* is the sample size, *E*_*i*_ equals one when *P*_*i*_*/y*_*i*_ >*z* and zero otherwise. This measurement of incidence does not take into account the distribution of catastrophic expenditure and so gives the same weight to households who incurred catastrophic expenditure regardless of ability to pay. A weighted incidence, *H*^*W*^ was thus used: *H*^*W*^ = *H*(1 − *C*_*E*_) where; *C*_*E*_ is the concentration index for *E*. *H*^*W*^ is the rank weighted incidence or head count and it takes into account the distribution of the catastrophic expenditure. The rank weighted head count puts a greater weight on the poor household that incur catastrophic payment than the rich. Thus, the *H* <*H*^*W*^. A negative *C*_*E*_ means that the poor are more likely to exceed the threshold than the rich.

The intensity of any catastrophic expenditure (i.e., the amount by which catastrophic expenditure exceeds the threshold) that might exist was measured as: $O=\frac{\sum_{i=1}^{N}O_{i}}{N}$ where $O_{i}=E_{i}\left[\frac{P_{i}}{y_{i}}-z\right]$. Again, to adjust for the distribution of catastrophic expenditure the rank weighted overshoot was used: *O*^*W*^ = *O*(1 − *C*_*O*_), where *C*_*O*_ is the concentration index for *O.* The mean positive overshoot (*MPO*) was also computed to provide information on the average overshoot among those who exceeded the threshold. $\text{MPO}=\frac{O}{H}$, thus *z* + *MPO* represents the average expenditure on premium as a share of ability to pay by those whose premium exceeded the threshold.

To find the characteristics of those who are likely to incur catastrophic expenditure multivariate logistic regressions were run: *E*_*i*_ = *α*_1_ + *α*_2_*X*_2_ + *α*_3_*X*_3_ + *α*_4_*X*_4_ + *α*_5_*X*_5_ + *e*_*i*_

where *X*_*2*_ is a vector of demographic characteristics: age and gender (female); *X*_*3*_ is a vector of variables for marital status, *X*_*4*_ is the ability to pay variable, and finally, *X*_*5*_ is the location dummy for Kumasi. Three regressions were run; one for each of the thresholds: *5*, *10*, and *15*.

## Results and discussion

### Data description

Data used for the study were collected from the administration of questionnaires on NHIS members randomly selected from the two main cities in the country: Accra (the capital city) and Kumasi (the commercial city). The reason for the choice of urban locations was due to the potential advantages. First, it is easier to get a large sample in an urban area than a rural area due to the dense population in the urban area. Second, the information on ability to pay which is important for the study is closely linked to the extent of subsistence level in lifestyle. High subsistence in the rural area can easily cause underestimation of ability to pay of rural households. For example, a farmer in a rural area may not spend as much on food as an urban dweller because the farmer may get some food supplies from the farm. The food expenditure is thus likely to be much higher for the urban dweller than the rural dweller. The respondents were household heads who paid premium for NHIS.

The population of Accra and Kumasi is more than four million making it impossible to use census data for the study. The choice of the sample size used for the study followed the steps in [19]. To ensure a sample size that predicts the population well, a sample error of ±3, a confidence interval of 95%, and a conservative variability of 50% - to incorporate the heterogeneity of the population in income distribution- were used. Hence the corresponding sample size according to the sample size Table in [19] cannot be less than 1,111. After the removal of observations with missing information, the sample size used then was 1,529.

Table 1 shows the descriptive statistics of the data used for the study. The average age of household heads was about 40 years and mostly educated. This is not a surprise because educated people are more likely to have a better understanding of the value of health insurance than the less educated or the uneducated. The household heads were mostly married and about 40% of them were females. Data on Accra formed 58% of the data. According to the NHIS 2009 report, the Greater Accra region has the lowest enrolment in the country. The reason for the lower enrolment is mostly because most of the workers in the formal sector have private health insurance provided by employers. Thus a large percentage of the enrollees in the Greater Accra region are likely to be from the informal sector.

**Table 1:** Data summary

**Variable**	**Percentage**
Age (average)	40.27 years
Premium	GHc 21.00 (US$13.13)
Females	40.5
Annual Expenditure on food and others	GHc 5,915.07 (US$3,696.91)
First Quintal	GHc 1,818.50 (US$1,136.56)
Second Quintal	GHc 3,574.81 (US$2,234.26)
Third Quintal	GHc 5,444.97 (US$3,403.11)
Fourth Quintal	GHc 7,530.99 (US$4,706.87)
Fifth Quintal	GHc 12,276.19 (US$7,672.62)
Accra	58.0
Education:	
· Tertiary	31.5
· Secondary	34.1
· Basic	30.2
· Illiterate	4.2
Marital Status	
· Married	68.7
· Divorced	10.5
· Widow/er	1.5
· Single	16.5
· Separated	2.6

The average annual premium per household was GHc21.00 (US$13.13) and the average annual expenditure on food and others was GHc5,915.07 (US$ 3,696.91). Thus, on average, the premium formed about 0.35% of the total ability to pay. However, the high variability in the ability to pay (standard deviation is 5119.5) and the lower variability in the premium (standard deviation is 15.67) implied that the proportion of premiums on ability to pay could be very high for some households. The five income groups show the extent of the variation in ability to pay. The poorest group (first quintal) had an average annual expenditure of GHc 1,818.50 (US$1,136.56) which implies that households in this group lived on US$3.11 a day. This amount was just above the poverty line of US$2.00 a day, indicating that indeed on average the indigent are not made to pay premium. However, with a standard deviation of 710.32, it is possible for the premium to be a burden to some of the poor or that some indigents were paying premium.

### Equity analysis

**Figure 1:**
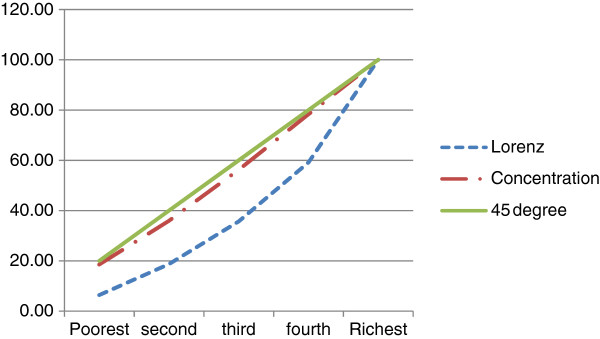
Measuring vertical equity for the premium.

Lorenz and concentration curves were drawn to find the progressivity of the premiums. The graph in Figure 1 shows that the concentration curve was everywhere greater than the Lorenz curve, an indication of the regressiveness of the premium contribution. The estimation of the Kakwani index produced an index of -0.32, as reported in Table 2. The negative sign indicates the regressiveness, hence confirming the results from the comparison of the concentration and Lorenz curves.

**Table 2:** Regression results of Kakwani indices

**Independent variables**	**Coefficient**	**P-value**	**Independent variables**	**Coefficient**	**P-value**
Constant	0.10	0.00	Constant	0.105	0.000
Fractional rank of			Fractional rank	−0.083	0.056
Ability to pay	−0.32	0.00	Tertiary	−0.013	0.580
			Secondary	0.003	0.898
			Female	0.001	0.938
			Kumasi	0.084	0.001
			married	0.051	0.005
			Tertiary*slope	−0.100	0.014
			Secondary*slope	−0.085	0.029
			Female*slope	−0.005	0.868
			Kumasi*slope	−0.249	0.000
			Married*slope	−0.092	0.004

To find out how regressivity varied across various categories of members another convenience regression for the Kakwani index was run with the inclusion of dummy variables for Kumasi, marital status, education, and gender. The results are reported in Table 2 and they show that the slope dummies were all negative and with the exception of gender all were also statistically significant. Such results imply that premiums were more regressive in Kumasi than Accra. The results also showed that regressivity was higher among the tertiary and secondary education than the basic and uneducated, and higher among the married than the unmarried. Gender however did not affect the regressivity.

The results on income redistribution effect of the premium had *V* as - 0.00113 which formed less than one percent (0.28%) of the total redistributive effect of the premium. This could be due to the small share of the premium in ability to pay. The *H* + *R* was 0.3933 forming 99.6% of the *RE.* Given that reranking is typically caused by horizontal inequity the sum was treated in the study as horizontal inequity. Thus the unequal treatment of equals was far more important in the redistributive effect of the premiums than the unequal treatment of unequals which has received attention in the literature.

It is not a surprise for the horizontal inequity to be important because the lack of information on ability to pay in the informal sector implies that the amount of premium paid could depend on factors other than ability to pay. To find out how the premium paid varied according to the characteristics of members, a linear regression was run with the natural log of premium as the dependent variable and the members’ characteristics: age, ability to pay variable, location, marital status, education, and gender. The natural log of premium was used because the functional form of a linear regression does not restrict the predicted values to positive and so it is possible to get negative predicted premiums hence weakening the results [20].

The results, reported in Table 3, show that premium increased slightly with ability to pay but fell for members in Kumasi as well as those with tertiary education. This implies that after controlling for ability to pay, people with tertiary education in a given income group were likely to pay a lower premium than those with lower level of education. The lower premium in Kumasi is not surprising because Kumasi has a lower cost of living than in Accra. More importantly, the low premium in Kumasi relative to Accra could explain why the percentage of NHIS enrolment in Kumasi is higher than that in Accra.

**Table 3:** Regression results on premium

**Independent variables**	**Coefficient**	**P-value**
Constant	2.934	0.000
Age	0.003	0.158
Ability to pay	0.0001	0.001
Number of adults	0.005	0.068
Tertiary	−0.182	0.000
Secondary	−0.042	0.330
Female	−0.041	0.225
Kumasi	−0.120	0.006
Married	−0.042	0.487

The results on catastrophic expenditure are reported in the Table 4. As expected, both the incidence and the intensity decreased with the threshold. The results show that the concentration indices were all negative implying that for any given threshold, the poor were more likely to incur catastrophic expenditure than the rich. This is consistent with the results on the regressiveness of the premium. The additional information here is that the regressiveness of the premium could be imposing financial burden on the poor. However, the results also show that the fraction of those that were likely to incur catastrophic payment formed less than 1.5% of the sample regardless of the threshold used. The fraction even reduced to less than 1% when the threshold increased from 5 to 10% and higher. This is consistent with the horizontal inequity meaning that some of the poor are made worse off by the premium. This implies that only a very small percentage of the sample make catastrophic payment. And this small percentage of the sample is likely to be poor.

**Table 4:** Incidence and intensity of catastrophic expenditure

	**5%**	**10%**	**15%**	**20%**
Incidence (*H*)	0.62%	0.41%	0.21%	0.14%
Standard deviation	0.078	0.064	0.045	0.037
Overshoot (*O*)	0.06%	0.03%	0.02%	0.01%
Standard deviation	0.010	0.007	0.005	0.003
Mean positive overshoot (*MPO*)	9.67%	7.31%	9.52%	7.14%
Average expenditure	14.67%	17.31%	24.52%	27.14%
Concentration index for *E*_*i*_ (*C*_*E*_)	−0.960	−0.955	−0.964	−0.954
Rank weighted incidence (*H*^*W*^)	1.22%	0.80%	0.41%	0.27%
Concentration index for *O* (*C*_*O*_)	−0.925	−1.106	−0.833	−0.932
Rank weighted overshoot (*O*^*W*^)	0.12%	0.06%	0.04%	0.02%

The results on the intensity of the catastrophic expenditure showed that the average degree by which the premium payment as a share of expenditure exceeded the threshold was less than one percent regardless of the threshold used. With the exception of the overshoot for the 5% threshold which was about 0.1%, the rest were all close to zero. The implication is that when spread over the whole sample, the payments were weakly catastrophic relative to the thresholds. However the mean overshoot among those whose payment exceeded the threshold (*MPO*) exceeded 7% regardless of the threshold. This implies that on average, those spending more than 5% of their ability to pay on premium on average spent 14.67% while those spending more than 20% on average spent 27.14%. The premium then imposes financial burden on a small minority of the poor.

The logistic regressions results are reported in Table 5. The results for the 20% threshold were not reported because they were very similar to those of 15% threshold. The results show that with the exception of ability to pay, none of the characteristics of the household head significantly affected the probability of incurring catastrophic expenditure. These results are consistent with the results that only a small minority of the people incurred catastrophic expenditure. Because only the ability to pay variable was statistically significant, a linear regression was run with the natural log of the ability to pay variable as the dependent variable and the characteristics of household head as the independent variables. The results are reported in the last column of Table 5 and they showed that ability to pay increased with the married household heads, age, number of adults in the household, and those in Kumasi. The results imply that households with these characteristics are not likely to incur catastrophic expenditures from the premium payment. These results confirm the conjecture that the NHIS enrolees in Accra are likely to be poor. The results also showed that people with secondary and basic education were likely to be poorer than the uneducated and those with tertiary education. The reason could be that people with little education (basic and/or secondary) may not be willing to take strenuous jobs that may be better paying than the ‘suitable’ jobs (such as receptionists, storekeeper, etc.) for the not so educated.

**Table 5:** Logistic regression results on factors affecting catastrophic expenditure

	**5%**	**10%**	**15%**	**Ability to pay**
Constant	−7.013	−23.450	28.354	7.604*
Age	0.006	−0.059	0.104	0.011*
Female	0.172	0.620	26.477	0.012
Ability to pay*	−0.003	−0.002	−0.022	
Number of adults	0.560	0.814	7.399	0.062*
Tertiary	5.358	7.192	−110.608	0.013
Secondary	3.983	6.515	−78.981	−0.279*
Basic	4.870	6.750	−72.999	-.251*
Kumasi	0.218	−0.544	5.662	0.704*
Married	0.311	15.596	8.730	0.183*

*Statistically significance at 5% significance level.

## Conclusion

The study has examined equity of the premium contribution of the Ghanaian National Health Insurance Scheme. The analysis focused on vertical and horizontal equity as well as the possibility of the premium imposing catastrophic expenditure on members. The results show that horizontal inequity dominates vertical inequity. Even though the premium is regersssive, the regressiveness does not affect redistribution of ability to pay as the horizontal inequity. After controlling for ability to pay, the study has shown that premiums are higher in Accra than Kumasi and lower among members with tertiary education than those without tertiary education. In addition the study has shown that the premium is likely to impose catastrophic expenditure on a small minority of the poor. Since universal coverage requires all residents having financial protection, having some residents, no matter how small the percentage, facing catastrophic expenditure can impede the achievement of universal coverage. The study makes three policy recommendations to improve equity.

Given that the indigent are to be exempted from the payment of premium, the result in the study could imply that it is possible for some indigent to be paying premium. Thus if extra efforts are made to identify the poor in the urban areas who qualify for exemption the percentage of those incurring catastrophic expenditure could be further reduced.

The reason for the large horizontal inequity could be due to the lack of uniform guidelines to Districts Mutual Health Schemes as to how much to charge according to characteristics that are related to the economic status of members. Such characteristics could include age, occupation, area of residence, housing, etc. For example, small restaurant owners, seamstresses/tailors, and hair salon owners could pay the same premium while gardeners, petty traders, and porters pay one premium. People in a similar occupation living in the same or similar residential area are also likely to be homogeneous in economic status. Following the Ghana Livelihood Empowerment program, the community could be used to confirm the economic status claimed by individuals. Certainly, such a program would not be without problems but would help improve significantly the current inequity problem.

Given that the premium payment imposes catastrophic expenditure on only a small minority of the members, it may not be necessary to cancel the premium as a source of revenue. Minimizing the catastrophic expenditure may be more beneficial than scraping the premium.

## Competing interest

The author declares that they have no competing interests.

## Author’s contribution

EA conceived the idea, ran the regressions and wrote the whole manuscript.
